# Will the adjustment of insertional pedicle screw positions affect the risk of adjacent segment diseases biomechanically? An in-silico study

**DOI:** 10.3389/fsurg.2022.1004642

**Published:** 2023-01-12

**Authors:** Chenyi Huang, Zongchao Liu, Zhangchao Wei, Zhongxin Fang, Zhipeng Xi, Ping Cai, Jingchi Li

**Affiliations:** ^1^ Department of Orthopedics, The Affiliated Traditional Chinese Medicine Hospital of Southwest Medical University, Luzhou, China; ^2^ Fluid and Power Machinery Key Laboratory of Ministry of Education, Xihua University, Chengdu, China; ^3^ Department of Spine Surgery, Jiangsu Province Hospital on Integration of Chinese and Western Medicine, Nanjing, China; ^4^ Department of Orthopedics, Affiliated Hospital of Nanjing University of Chinese Medicine, Nanjing, China

**Keywords:** adjacent segment diseases, biomechanical deterioration, insertional screw positions, arm of force, pedicle screw fixation

## Abstract

**Background:**

The fixation-induced biomechanical deterioration will increase the risk of adjacent segment diseases (ASD) after lumbar interbody fusion with Bilateral pedicle screw (BPS) fixation. The accurate adjustment of insertional pedicle screw positions is possible, and published studies have reported its mechanical effects. However, no studies clarified that adjusting insertional screw positions would affect the postoperative biomechanical environment and the risk of ASD. The objective of this study was to identify this issue and provide theoretical references for the optimization of insertional pedicle screw position selections.

**Methods:**

The oblique lumbar interbody fusion fixed by BPS with different insertional positions has been simulated in the L4-L5 segment of our previously constructed and validated lumbosacral model. Biomechanical indicators related to ASD have been computed and recorded under flexion, extension, bending, and axial rotation loading conditions.

**Results:**

The change of screw insertional positions has more apparent biomechanical effects on the cranial than the caudal segment. Positive collections can be observed between the reduction of the fixation length and the alleviation of motility compensation and stress concentration on facet cartilages. By contrast, no pronounced tendency of stress distribution on the intervertebral discs can be observed with the change of screw positions.

**Conclusions:**

Reducing the fixation stiffness by adjusting the insertional screw positions could alleviate the biomechanical deterioration and be an effective method to reduce the risk of ASD caused by BPS.

## Introduction

The bilateral pedicle screw (BPS) is extensively used in spinal operations to restore physiological alignment, maintain stability, and correct hypermotility (1, 2). During the lumbar interbody fusion surgery (LIF), BPS could construct three-column instant stability by transpedicular fixation (2, 3). Although BPS is the gold standard of additional fixation technique in LIF surgery, the stiffness-increasing mechanism of BPS will lead to the fixation-induced pathological load transmission pattern (e.g., stress concentration and motility compensation in adjacent segments) and resulting adjacent segment diseases (ASD) (2–4).

Surgeons advocate removing BPS after solid interbody fusion, and its positive biomechanical effects on adjacent segments have been reported by in-silico mechanical simulations (2, 3). But this method has not been widely promoted in clinical practice, for it is difficult for patients without severe symptoms to accept a second surgical trauma. Additionally, low stiffness material connection rods (e.g., Polyether ether ketone rod) have been designed to alleviate postoperative biomechanical deterioration and reduce the risk of ASD. Their biomechanical advantages have also been proved by in-silico and in-vitro mechanical studies (4–8). However, a higher incidence rate of fixator failure and revision surgery inhibits the promotion of these instrumentations (8, 9). Hence, BPS is still the gold standard of the additional fixation device in LIF at the present stage. The optimization of surgical procedures during the use of BPS, rather than the replacement of BPS, may have a better clinical application prospect. During the modification of the screw trajectory, studies reported that the shift of the screw insertion point medially in the coronal plane could alleviate biomechanical deterioration and reduce the risk of ASD (10, 11); the biomechanical mechanism behind the relation between these screw insertion techniques and the risk of ASD should include not only biomechanical changes caused by different grades of the violation of zygapophyseal joints (ZJ) (12, 13), but also the change of fusion segmental stiffness and resulting overall lumbar biomechanical changes (2, 14).

The percutaneous BPS insertion technique is widely promoted (15, 16). Its insertional screw positions can be accurately adjusted under the guidance of the C-arm, but no studies were elucidating the biomechanical changes with the adjustment of screw insertion positions in the sagittal plane. Adjusting screw insertion positions will affect the BPS's fixation length and locally biomechanical impacts on adjacent segments (2, 14). Considering long segment LIF with the expansion of fixation length has been proven a risk factor of ASD (17, 18), we believe that optimizing screw insertion positions in the sagittal plane (i.e., reducing the fixation length) may be an effective method to reduce the risk of ASD biomechanically. The objective of this study was to identify the biomechanical significance of insertional pedicle screw positions on the risk of ASD. Published literature has not adequately clarified this issue to the best of our knowledge.

## Methods

### Model construction

We simulate oblique lumbar interbody fusion (OLIF) fixed by BPS with different insertional positions in a previously constructed and well-validated lumbosacral model (19, 20). Bone structures include cortical, cancellous, and bony endplates (BEP), the thickness and morphology of BEPs were defined according to the measurement of large sample imaging data (21, 22). Nonbony components include the intervertebral disc (IVD) and ZJ cartilages. IVD consists of the nucleus core, the surrounding annulus, and cartilage endplates (CEP) on the cranial and caudal sides of the nucleus and inner part of the annulus (23, 24).

### Boundary and loading conditions

Models were computed under identical loading conditions, including flexion, extension, bending, and rotation. Sizes of the moment in the mechanical indicators computation process were consistent with the validation of the range of motion (ROM). They were set to be symmetric in the sagittal plane to increase their computational efficiency by allowing the unilateral calculation of the bending and axial rotation loading conditions (19, 20). Hybrid elements (including tetrahedron and hexahedron) with different mesh sizes were established in different components, and smaller mesh sizes were used in structures with low thickness and large deformation (20, 25).

In the definition of material properties, cortical and cancellous bone were set as anisotropic materials (26, 27), other parts of the model were defined by isotropic law (26, 27). The annulus was assumed to be hypoelastic (26, 28), and the nucleus was set as an incompressible “semi-fluid pad” (25, 29). Ligaments structures and capsules of ZJ were defined as cable elements in the pre-processing step of FEA (Table 1) (25, 29–31). Contact elements defined facet cartilages of ZJ, and its frictional coefficient was set as zero (29, 32).

**Table 1:** Material properties of components in current models.

Components	Elastic modulus (MPa)	Poisson's ratio	Cross-section (mm^2^)
Cortical	*E_xx_*_ _= 11,300	*V_xy_*_ _= 0.484	/
	*E_yy_*_ _= 11,300	*V_yz_*_ _= 0.203	
	*E_zz_*_ _= 22,000	*V_xz_*_ _= 0.203	
	*G_xy_*_ _= 3,800		
	*G_yz_*_ _= 5,400		
	*G_xz_*_ _= 5,400		
Cancellous	*E_xx_*_ _= 140	*V_xy_*_ _= 0.45	/
	*E_yy_*_ _= 140	*V_yz _*= 0.315	
	*E_zz_*_ _= 200	*V_xz_*_ _= 0.315	
	*G_xy_*_ _= 48.3		
	*G_yz_*_ _= 48.3		
	*G_xz_*_ _= 48.3		
Bony endplates	12,000	0.3	/
Annulus	Hypoelastic material		/
Nucleus	1	0.49	/
Cartilage endplates	10	0.4	/
Anterior longitudinal ligaments	Calibrated load-deformation curved under different loading conditions	0.3	60
Posterior longitudinal ligaments	Calibrated load-deformation curved under different loading conditions	0.3	21
Ligamentum flavum	Calibrated load-deformation curved under different loading conditions	0.3	60
Interspinous ligaments	Calibrated load-deformation curved under different loading conditions	0.3	40
Supraspinous ligaments	Calibrated load-deformation curved under different loading conditions	0.3	30
Intertransverse ligaments	Calibrated load-deformation curved under different loading conditions	0.3	10
Capsular	7.5 (\25%)	0.3	67.5
	32.9 ([25%)		
PEEK	3,500	0.3	/
Titanium alloy	110,000	0.3	/

### Model calibration and validation

All freedom degrees were fixed under the inferior surfaces of current models, and moments were applied on their superior surfaces (5, 29). The stiffness of ligaments under different loading conditions was calibrated to reduce the difference between the computed ROM in the L4-L5 segment and in-vitro studies (33, 34). A mesh convergency test on the intact model was performed by evaluating intradiscal pressure (IDP) change with different mesh sizes. The model was considered converged if the change of computed IDP was less than 3% (35, 36), multi-indicators model validation has been accomplished by comparing the computed ROM, IDP, the disc compression (DC), and the facet contact force with values from in-vitro studies under different sizes and directions load to ensure computational credibility (37, 38).

### Surgical simulations and ASD's risk evaluation

The L4-L5 segment has been selected to simulate the oblique lumbar interbody fusion (OLIF) fixed by BPS with different insertional positions for the incidence rate of lumbar degenerative diseases in this segment was higher than that of the L3-L4 segment, and the L5-S1 segment was not suitable for OLIF generally (15, 16). Lateral parts of the annulus, all of the nucleus, and CEPs in the surgical segment were removed, and a PEEK OLIF cage (18 mm long and 50 mm wide) filled with grafted bony material was inserted into interbody space (15, 39). It was assumed that the disc height and lordotic angle of disc space were not affected by cage insertion, and the outline between cage and BEP was assumed to be perfectly matched (3, 30, 40). Considering ASD was a typical long-term complication, the boundary conditions have been defined to simulate solid interbody fusion. In which, the contact type between grafted bone and BEP was set to be “bounded” (completely constrains the motion under all degrees of freedom), and the frictional coefficient in surfaces between cage and BEP was 0.8 (41, 42).

During the simulation of titanium alloy (Ti6Al4V) BPS fixation with different insertion positions, bilateral pedicle screws (were inserted into L4 and L5 vertebral bodies. The axes of screws on the cross-section were parallel to the pedicle axis, and the screw axis was parallel to which of corresponding cranial BEP (5, 16). The connection between the screw tulip and the nut was simplified to reduce the computational burden (2, 5). Five postoperative models with different insertional screw positions have been constructed, and the screw compaction effect was simulated by adjusting the material property of bony tissue around the screw thread (43, 44). Motility parameters, stress distribution in IVD, and ZJ in both cranial and caudal sides of functional units were recorded to evaluate the risk of ASD.

## Results

### Multi-indicators model validation

**Figure 1:**
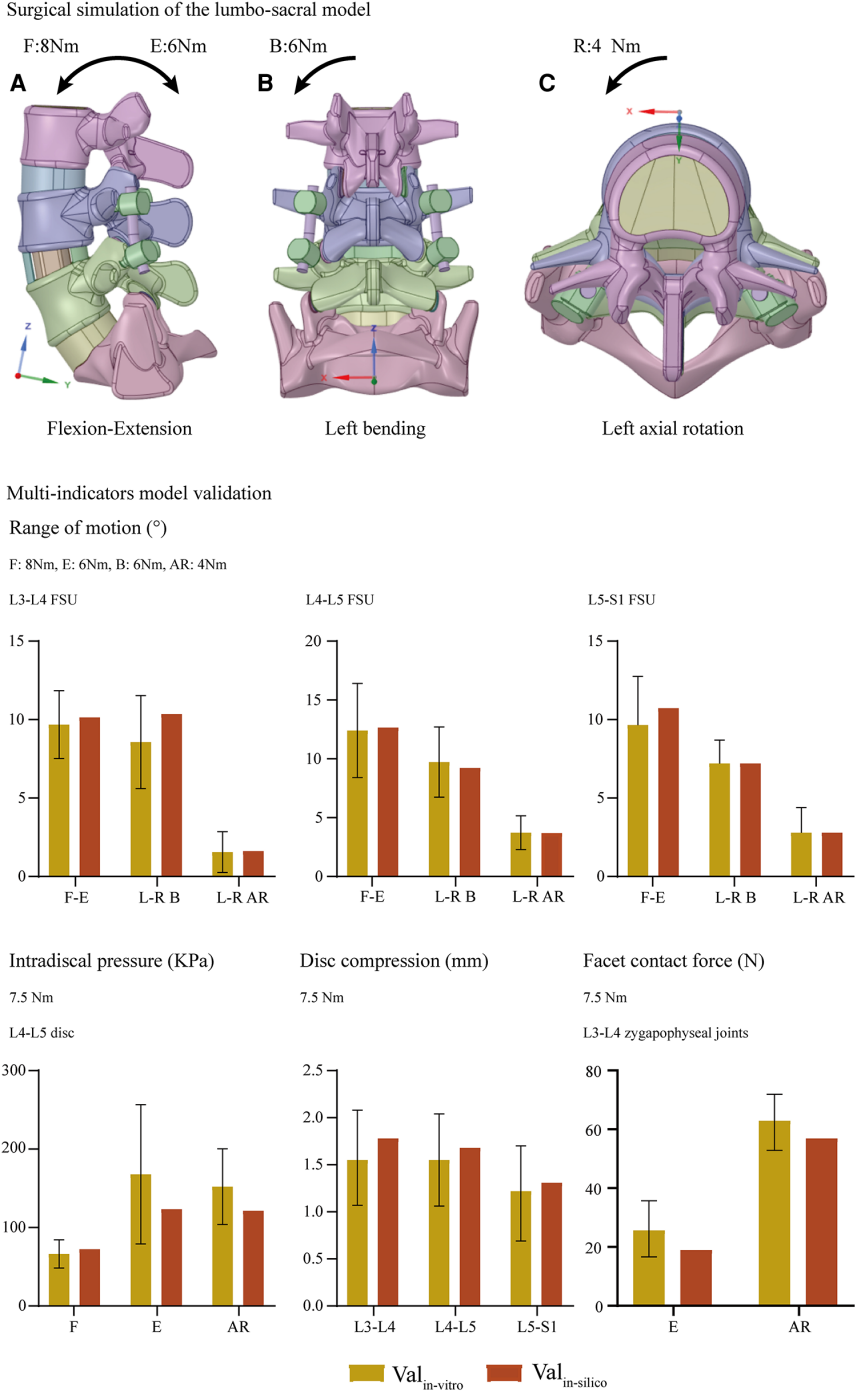
Surgical simulations and multi indicators model validation: Val_in−vitro_, indicators measured by published in-vitro studies; Val_in−silico_, indicators computed by the current in-silico study; F-E, flexion-extension; L-R, left-lateral; B, bending; AR, axial rotation.

Well-validated computational results can be recorded in the intact model. Specifically, the values of computed ROM and DC under were compared with which in in-vitro studies reported by Renner et al (38), values of IDP were compared with the study published by Schilling et al (7), and which of FCF were also compared with Wilson et al.'s study (37). These indicators computed by the intact model were within ±1 standard deviation of the average values reported by the above-mentioned in-vitro studies, proving that current models could make a good representation of real biomechanical situations (Figure 1).

### Changes in mechanical indicators related to ASD

**Figure 2:**
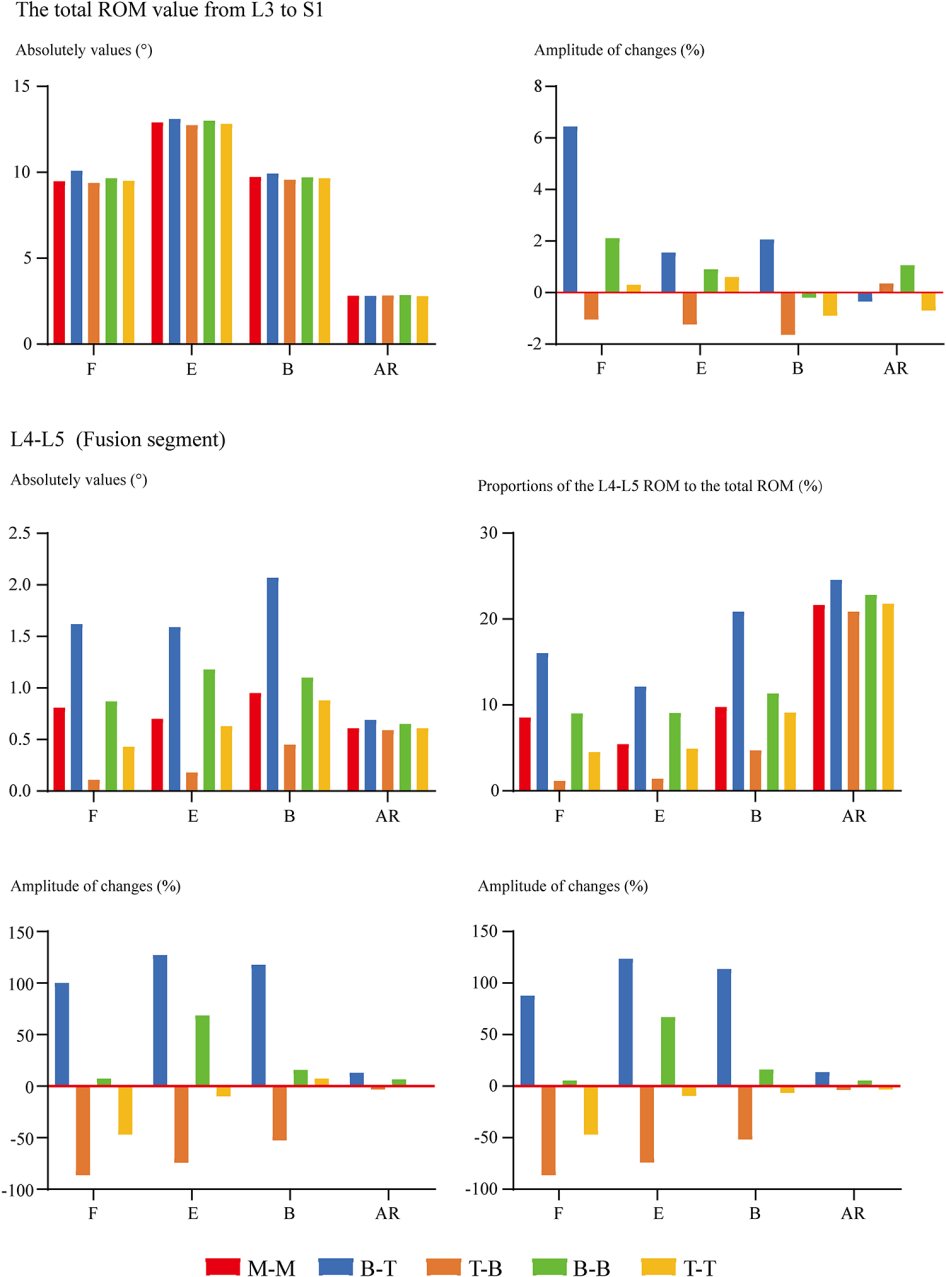
Comparison of overall and surgical segment's ROM between surgical models with different insertional screw positions. F, Flexion; E, Extension; B, Bending; AR, Axial rotation.

**Figure 3:**
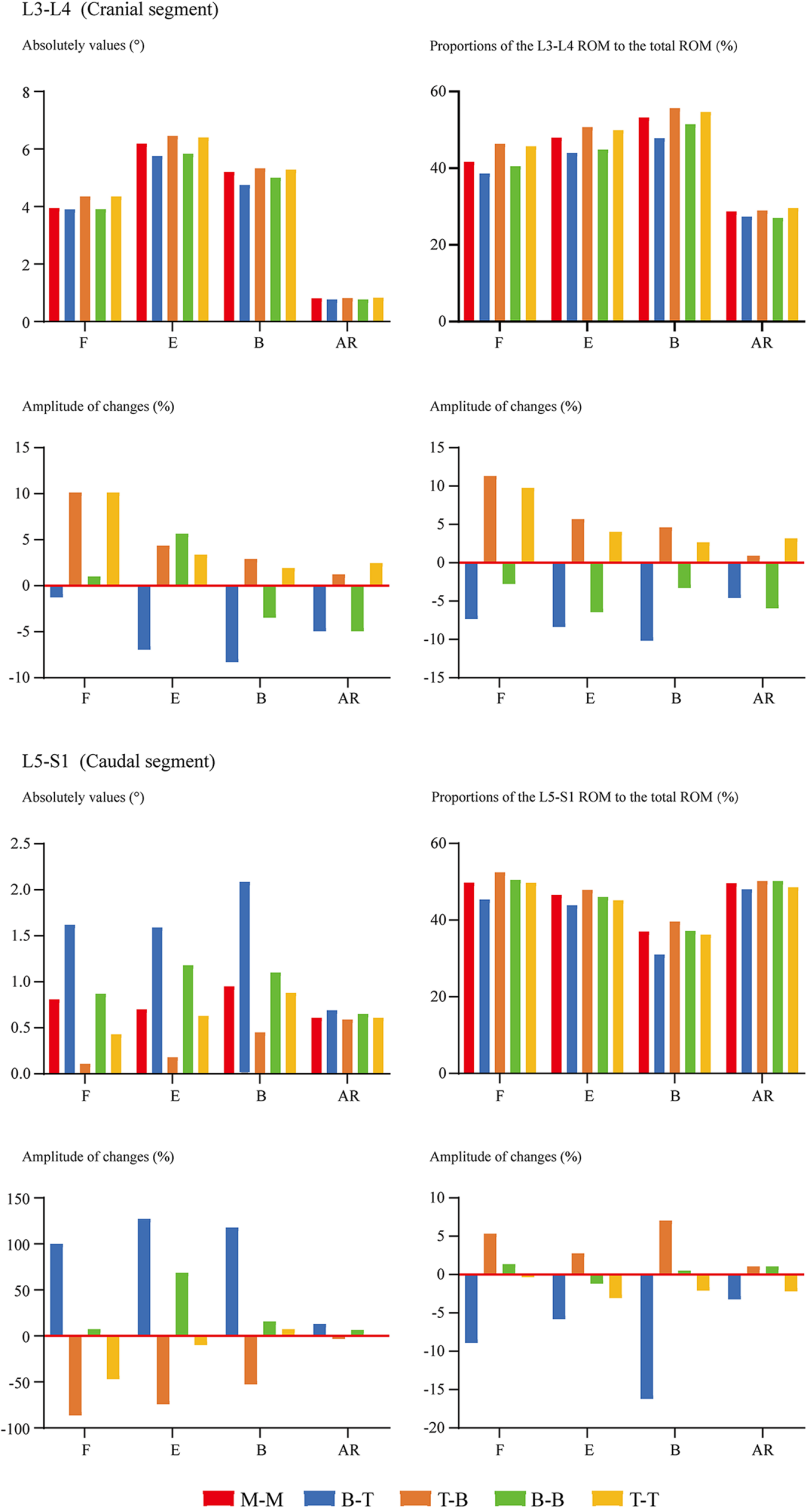
Comparison of cranial and caudal adjacent segments’ ROM between surgical models with different insertional screw positions. F, Flexion; E, Extension; B, Bending; AR, Axial rotation.

Overall ROM, ROM in different segments (including the surgical and adjacent segments), and the proportion of different segmental ROM to the overall value have been computed and recorded to evaluate the motility compensation. Except for the axial rotation condition, positive relations between BPS's fixation length and fixational stiffness can be observed. Specifically, the change of fixation length will slightly affect the overall ROM (the variation range was smaller than 5% except for model 2 (model with shortest fixation length) under the flexion loading condition). By contrast, the change of ROM in the fusion segment was dramatically under most loading conditions. Meanwhile, pathological motility compensation could be amplified and alleviated by increasing and decreasing the arm of force in these segments, especially under the flexion condition in the cranial and bending in the caudal segment (Figures 2, 3).

**Figure 4:**
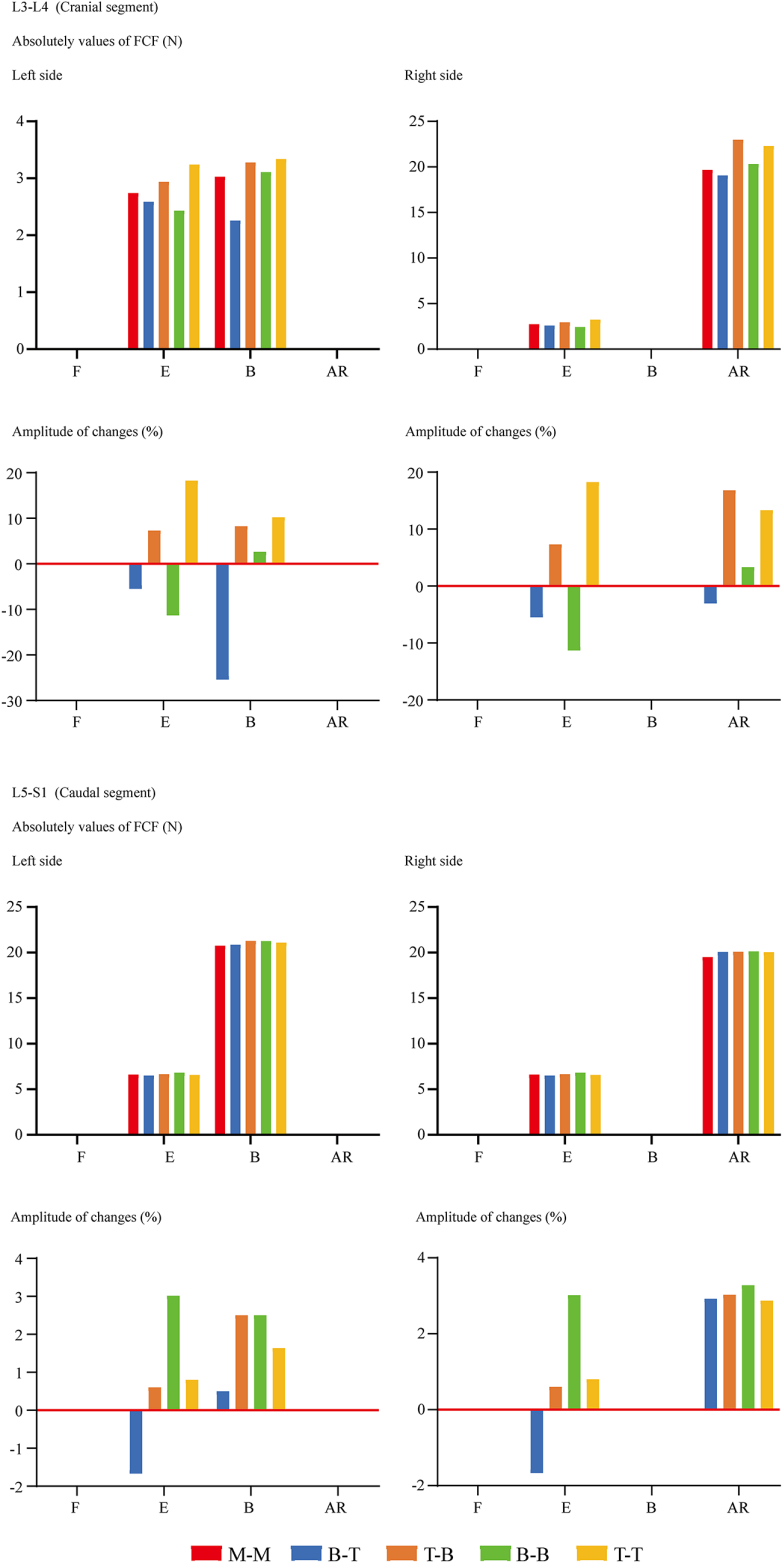
Comparison of FCF between surgical models with different insertional screw positions. F, Flexion; E, Extension; B, Bending; AR, Axial rotation.

**Figure 5:**
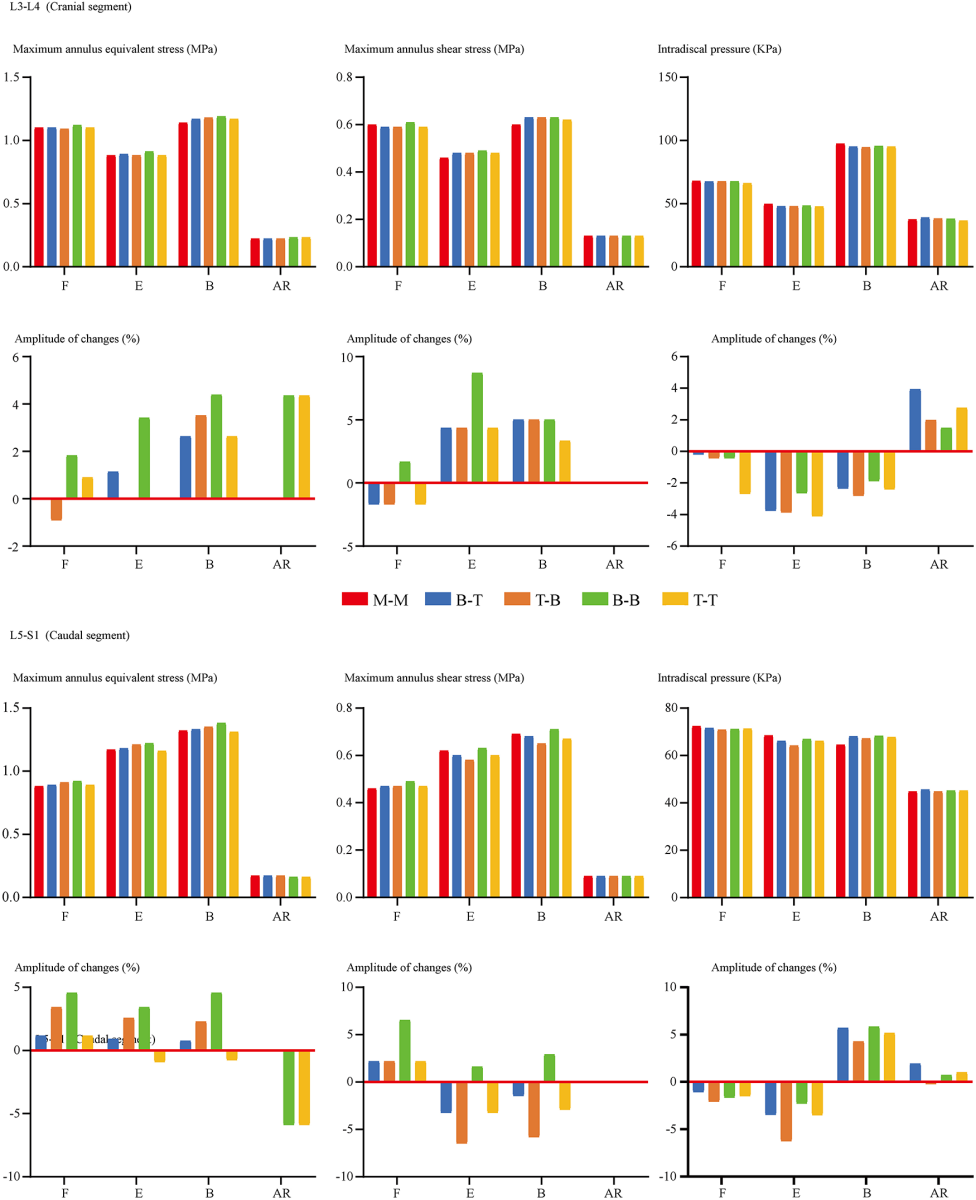
Comparison of indicators related to the cranial and caudal disc degeneration between surgical models with different insertional screw positions. F, Flexion; E, Extension; B, Bending; AR, Axial rotation.

FCF was not recorded under the flexion loading condition for ZJ cartilages that were not in contact. For the same reason, FCF on the opposite side to the bending condition and the rotation side could not be recorded. In other words, FCF under left lateral bending is observed on left-side cartilages, while FCF under left axial rotation is observed on right-side cartilages. The change of insertional screw positions can lead to the change of FCF. Generally, reducing the arm of force in adjacent segments will decrease FCF and vice versa (Figure 4). The variation tendency of the cranial side was more pronounced than the caudal one. To investigate the risk of disc degeneration, we calculate IDP, maximum values of annulus shear and equivalent stress (Figure 5). Inconsistent with the variation tendency of ROM and FCF, no apparent tendency of these mechanical indicators can be observed with the change of insertional screw positions, especially under the rotation condition.

## Discussion

This work evaluated biomechanical deterioration and the related risk of ASD after OLIF fixed by BPS with different insertional screw positions. An intact lumbosacral model and corresponding OLIF models were constructed, and biomechanical indicators closely related to ASD were computed and evaluated. The importance of the biomechanical environment for achieving positive postoperative clinical outcomes has been repeatedly demonstrated (2, 17, 29). Thus, investigations on the biomechanical effects of different insertional screw positions are of great significance for optimal operative strategy and reducing the risk of ASD.

**Figure 6:**
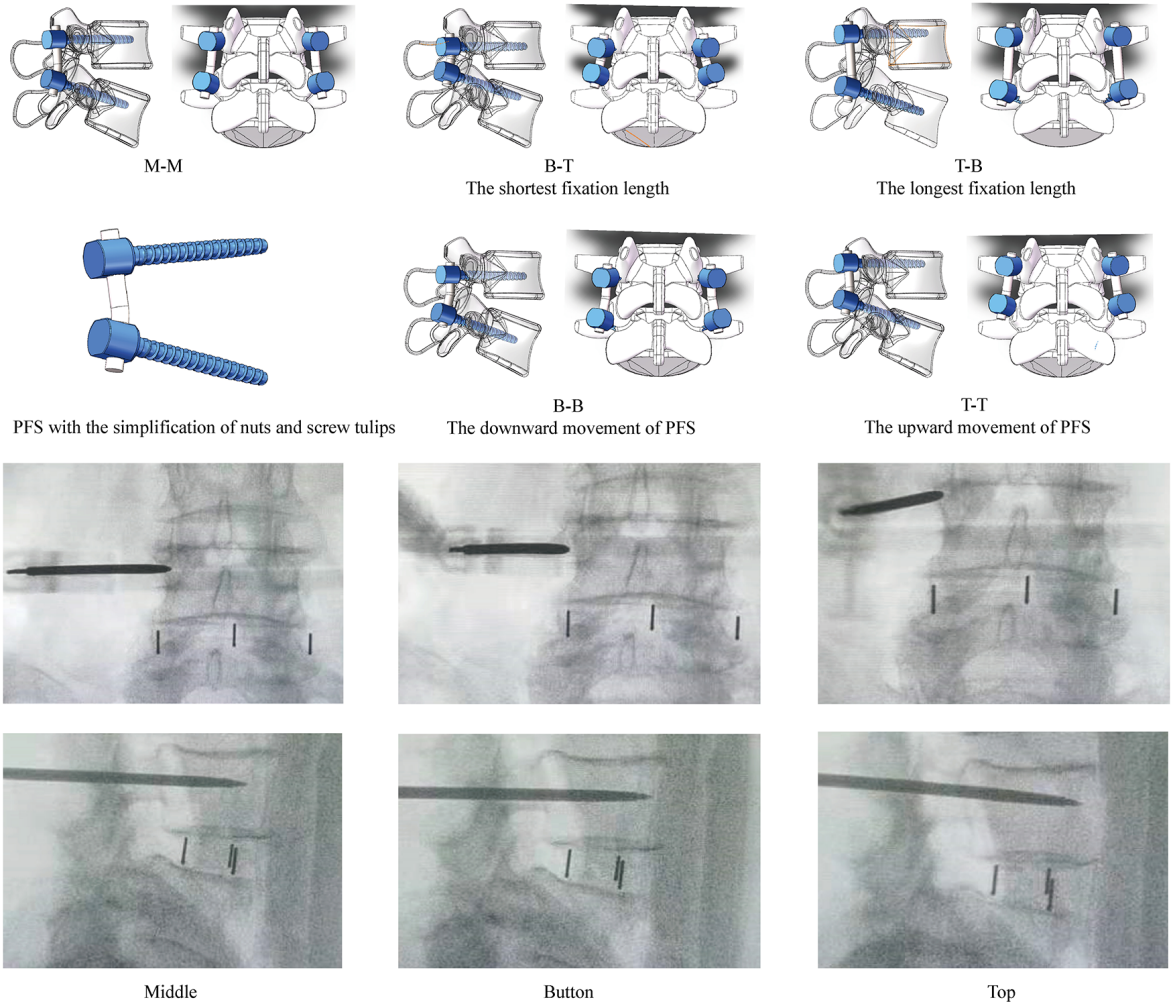
Diagrams of OLIF fixed by PFS with different insertional screw positions, and the highly adjustable of percutaneous BPS insertion. M-M: Screws were inserted into the middle positions of both cranial and caudal vertebral bodies; B-T: Screws were inserted into the bottom of the cranial and the top of the caudal vertebral bodies (Shortest fixation length of PFS); T-B: Screws were inserted into the top of the cranial and the bottom of the caudal vertebral bodies (Longest fixation length of PFS); BB: Screws were inserted into the bottom of both cranial and caudal vertebral bodies (The downward movement of PFS); TT: Screws were inserted into the top of both cranial and caudal vertebral bodies (The upward movement of PFS).

OLIF, rather than other LIF operations, has been selected for the following reasons. The percutaneous pedicle screw insertion was accomplished under C-arm fluoroscopy in OLIF, and the adjustment of insertion positions is feasible in this operation (Figure 6). By contrast, selecting screw insertion positions in other lumbar fusion operations (e.g., transforaminal and posterior lumbar interbody fusion) was based on identifying anatomic structures (10, 11). Considering the prevalence of anatomic variations and the hypertrophy of the articular process during the pathological process of spinal stenosis (45, 46), it is difficult to accurately judge and adjust the exact insertional screw position under the freehand pedicle insertion process. Furthermore, for the same reason, the promotion of the optimized insertional screw positions elucidated by this study may also be limited in LIF fixed by percutaneous pedicle screw.

The deterioration of the biomechanical environment caused by inappropriate surgery may be continuously amplified and lead to a devastating prognosis (17, 19, 47). Therefore, optimizing a surgical technique based on a biomechanical study is significant. There are three common pathological changes of ASD: disc degeneration, ZJ degenerative osteoarthritis, spinal stenosis, and segmental instability (17, 48). The annulus-driven phenotype is the most common reason for disc degeneration in the lower lumbar spine (49, 50). Stress concentration on the annulus, especially on the post and post-lateral parts of the annulus, were related to different types of annulus tears (34, 51). Meanwhile, the aberrant increase of IDP could also increase the risk of annulus failure (26, 52); therefore, annulus stress distribution and IDP are critical indicators in related mechanical studies (26, 53). Simultaneously annulus tears and increased intradiscal pressure would promote disc herniation. The in-growth of blood vessels along annulus tears will promote the inflammatory response, leading to extracellular matrix catabolism and further disc degeneration (50, 54). The in-growth of pain-sensing nerve fibers is also the primary reason for postoperative pain recurrence in ASD (54, 55).

The pathological change in ASD was not limited to IVD. The degenerative osteoarthritis, hypertrophy of the articular process, and resulting spinal canal stenosis were also essential triggers symptoms recurrence (42, 56). Therefore, ZJ degeneration should also be considered in ASD, which can be well reflected by evaluating the FCF (19, 26) Additionally, as mentioned above, postoperative pathological motility compensation and resulting spinal instability is also a basic form of ASD (17, 42), which could be reflected by the variation of ROM and its proportion (5, 47). Therefore, ROM can be used as an indicator for model calibration and validation, and assess ASD's risk. Moreover, the interaction between segmental instability and spinal canal stenosis was also clearly elucidated, reactive hyperplasia of the articular process and ligamentum structures caused by segmental instability was the main reason for spinal stenosis over a long period (45, 57). In a word, by computing these biomechanical indicators, the risk of ASD could be investigated systematically.

Based on the current computational results, slight changes in stress concentration on the disc can be observed in cranial and caudal IVDs. Therefore, we can deduce that the tendency of disc degeneration acceleration may not be changed obviously with the change of arm of force. By contrast, the fixation stiffness in the surgical segment and motility compensation in adjacent segments could be distinctly affected by the change of fixation length. Pronounced motility compensation can be recorded when the fixation length increases and the BPS shift towards the measured side. Meanwhile, although the range of variations is higher in the cranial than the caudal segment, the overall variation tendency of FCF is still consistent with which of the motility compensation. Considering above mentioned interaction between segmental instability and spinal canal stenosis (45, 57), the reduction of BPS's fixation length by adjusting the percutaneous BPS's positions could optimize the local biomechanical environment and reduce the risk of adjacent segmental instability in the short term and spinal stenosis in the long term in both cranial and caudal motion segments adjacent to the surgical segment with percutaneous BPS fixation.

Admittedly, the current study results should be interpreted within the context of the following-mentioned limitations. Firstly, the mechanical effect of ligaments can only be acted on artificially selected positions rather than their entire original surfaces. We defined these ligaments as cable elements, and the potential risk of mechanical indicators distortions should be considered. However, we believe that the computational results elucidated by current models are still reliable for the following reasons. The definition of cable ligaments has been widely used in the same kind of in-silico spinal studies (25, 29, 51), and the multi-indicators model validation has guaranteed the credibility of the current models. Additionally, no attach positions of cable elements are defined on structures with computed indicators (e.g., annulus, CEPs, and facet cartilages of ZJ); this determines that even if there is computational distortion, it can be excluded from the indicator's computation. The definition of ligaments should still be optimized in future in-silico studies. Meanwhile, the damage to facet joint capsule and facet cartilages were not simulated in this study. That's because current FEA studies never research this topic, so it is not easy to find a widely accepted standard to simulate this topic in our models. But the simulation of this topic may be necessary and should be performed in our future studies. Moreover, we could not provide clinical evidence to verify the computed biomechanical changes in the current study. We admit that corresponding clinical evidence is of great significance to this topic, and we will try to provide clinical evidence in our future studies.

## Conclusion

Collectively, computed indicators in this study elucidated that during LIF operations fixed by percutaneous BPS, reducing the fixation stiffness by adjusting the insertional screw positions on the sagittal plane could alleviate motility compensation and stress concentration on ZJ cartilages, especially on the cranial segment. Thus, this mechanical effect may be an effective method to reduce the risk of ASD (adjacent segmental instability in the short term and spinal stenosis in the long term).

## Data Availability

The original contributions presented in the study are included in the article/Supplementary Material, further inquiries can be directed to the corresponding author/s.
